# Licochalcone A Inhibits the Proliferation of Human Lung Cancer Cell Lines A549 and H460 by Inducing G2/M Cell Cycle Arrest and ER Stress

**DOI:** 10.3390/ijms18081761

**Published:** 2017-08-12

**Authors:** Chenyu Qiu, Tingting Zhang, Wenxin Zhang, Lina Zhou, Bin Yu, Wei Wang, Zhihong Yang, Zhiguo Liu, Peng Zou, Guang Liang

**Affiliations:** Chemical Biology Research Center, School of Pharmaceutical Sciences, Wenzhou Medical University, Wenzhou, Zhejiang 325035, China; wenzhouqiuchenyu@163.com (C.Q.); Ting920236@163.com (T.Z.); m13676551640@163.com (W.Z.); zhoulina1026@163.com (L.Z.); wenzhoubinyu@163.com (B.Y.); wangwei941226@163.com (W.W.); wenzhouyangzhihong@163.com (Z.Y.); wzmcliangguang@163.com (G.L.)

**Keywords:** Licochalcone A, cell cycle arrest, ER stress, lung cancer, apoptosis

## Abstract

Licochalcone A (LicA), a flavonoid isolated from the famous Chinese medicinal herb Glycyrrhiza uralensis Fisch, has wide spectrum of pharmacological activities. In this study, the anti-cancer effects and potential mechanisms of LicA in non-small cell lung cancer (NSCLC) cells were studied. LicA decreased cell viability and induced apoptosis in a dose-dependent manner in NSCLC cells. LicA inhibited lung cancer cells growth by blocking cell cycle progression at the G2/M transition and inducing apoptosis. LicA treatment decreased the expression of MDM2, Cyclin B1, Cdc2 and Cdc25C in H460 and A549 cancer cell lines. In addition, LicA induced caspase-3 activation and poly-ADP-ribose polymerase (PARP) cleavage, which displayed features of apoptotic signals. Furthermore, LicA increased the expression of endoplasmic reticulum (ER) stress related proteins, such as p-EIF2α and ATF4. These data provide evidence that LicA has the potential to be used in the treatment of lung cancer.

## 1. Introduction

Non-small cell lung cancer (NSCLC) is a leading cause of cancer-related deaths worldwide [1]. The current pathological classification of NSCLC identifies three main histological subtypes including squamous cell carcinoma, adenocarcinoma, and large cell carcinoma. Surgery is the main therapeutic option [2]. Most of NSCLC patients present with advanced disease upon diagnosis and the therapeutic strategy for these patients is drug therapy. Targeted cancer therapies have produced significant clinical responses in NSCLC patients; for example, epidermal growth factor receptor tyrosine kinase inhibitors (erlotinib, gefitinib, and osimertinib) have been successfully used for NSCLC patients with EGFR mutation [3,4,5,6]. However, more than half of NSCLC patients are harboring wild type EGFR for whom the treatment strategies are docetaxel- or cisplatin-based chemotherapy [7,8,9]. Due to the obvious severe side effects and drug resistance of docetaxel or cisplatin, there is an urgent need to search for novel and safe anti-cancer agents.

Natural products have historically been invaluable as a source of therapeutic agents. Many anti-cancer agents, such as vincristine [10], paclitaxel [11], and etoposide [12], are naturally derived and play a critical role in chemotherapy. In previous research, we found that piperlongumine, a natural alkaloid isolated from the fruit of long pepper, selectively kills gastric cancer cells while sparing their normal counterparts [13]. In addition, we clarified that two curcumin analogues selectively kill gastric cancer cells via activating ER stress pathway [14,15].

Licochalcone A (LicA) is a flavonoid extracted from licorice root, presents a wide range of pharmacological effects, such as anti-cancer [16,17], anti-inflammation [18], and anti-bacterial [19]. The anti-cancer effect of LicA has been demonstrated in diverse types of cancer cells, including gastric cancer AGS cells [20], hepatocellular carcinoma HepG2 cells [21], as well as ovarian cancer OVCAR-3 and SK-OV-3 cells [22]. Several in vivo studies have indicated that LicA presents remarkable therapeutic effects for gastric cancer [23], cervical cancer [17], and colon cancer [24]. Moreover, LicA obviously reduced the cisplatin-induced kidney damage without affecting its anti-cancer effects. In the present study, we have examined the effect of LicA in lung cancer cells in vitro. We show that LicA induces cell cycle arrest and apoptosis in lung cancer cells. We also demonstrate that LicA activates the endoplasmic reticulum (ER) stress pathway. Our study indicates that LicA could be a potential candidate for the treatment of human lung cancer.

## 2. Results

### 2.1. LicA Effectively Suppressed the Proliferation of Human Lung Cancer Cells

**Figure 1:**
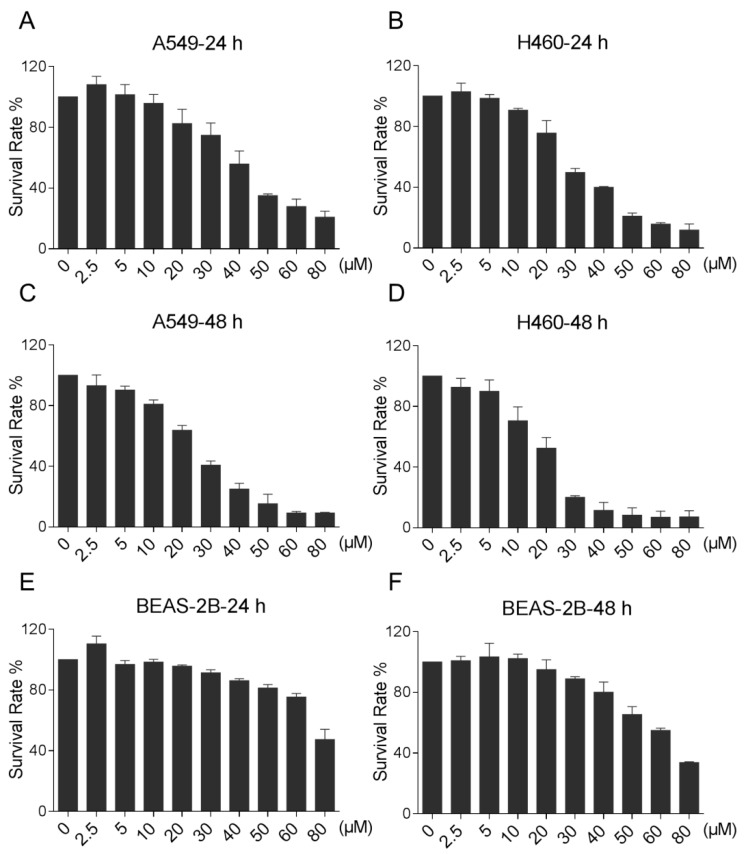
LicA inhibits lung cancer cells growth. (**A**–**F**) The effect of LicA on the proliferation of human lung cancer cells and normal cells. A549, H460 or BEAS-2B cells were incubated with increasing doses of LicA (2.5, 5, 10, 20, 30, 40, 50, 60 and 80 μM) for 24 or 48 h respectively. Cell viability was determined by MTT assay. All images shown here are representative of three independent experiments with similar results.

We first determined the effect of LicA on the cell viability of two human lung cancer cell lines, A549 and H460, by MTT assay. As shown in Figure 1A–D, LicA treatment significantly decreased the viability of A549 and H460 cells in a dose-dependent manner. For instance, 40 μM of LicA suppressed the growth of lung cancer cells by 45–80% upon 24 or 48 h of treatment. Moreover, LicA showed a low cytotoxicity on normal human lung epithelial cells (Figure 1E,F). These results suggest that LicA may be a potential candidate for the treatment of lung cancer.

### 2.2. LicA Induced G2/M Cell Cycle Arrest in Human Lung Cancer Cells

**Figure 2:**
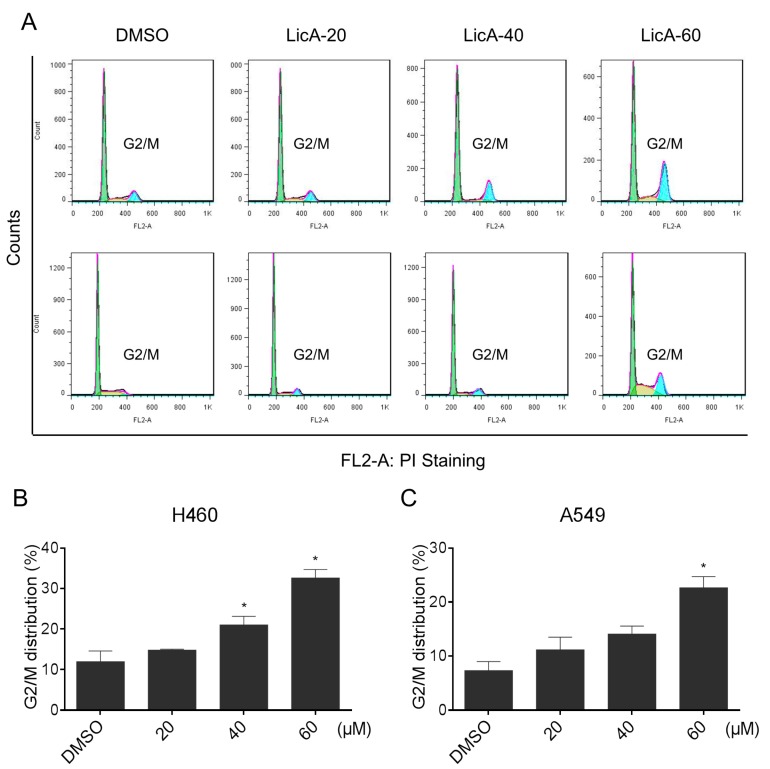
LicA induces cell cycle arrest in human lung cancer cells. (**A**–**C**) H460 and A549 cells were treated with LicA (20, 40 or 60 μM) for 16 h, and the cell cycle distribution was analyzed by flow cytometry. Histogram illustrating of the rate of G2/M phases cells from flow cytometry analysis of three separate treatments (* *p* < 0.05). All images shown here are representative of three independent experiments with similar results.

To determine whether the growth inhibition of lung cancer cells by LicA was caused by cell cycle arrest, two lung cancer cell lines were treated with various concentrations of LicA for 16 h, then the cell cycle was determined by flow cytometry. The results in Figure 2A–C showed that LicA dose-dependently induced G2/M arrest in the two lung cancer cells.

**Figure 3:**
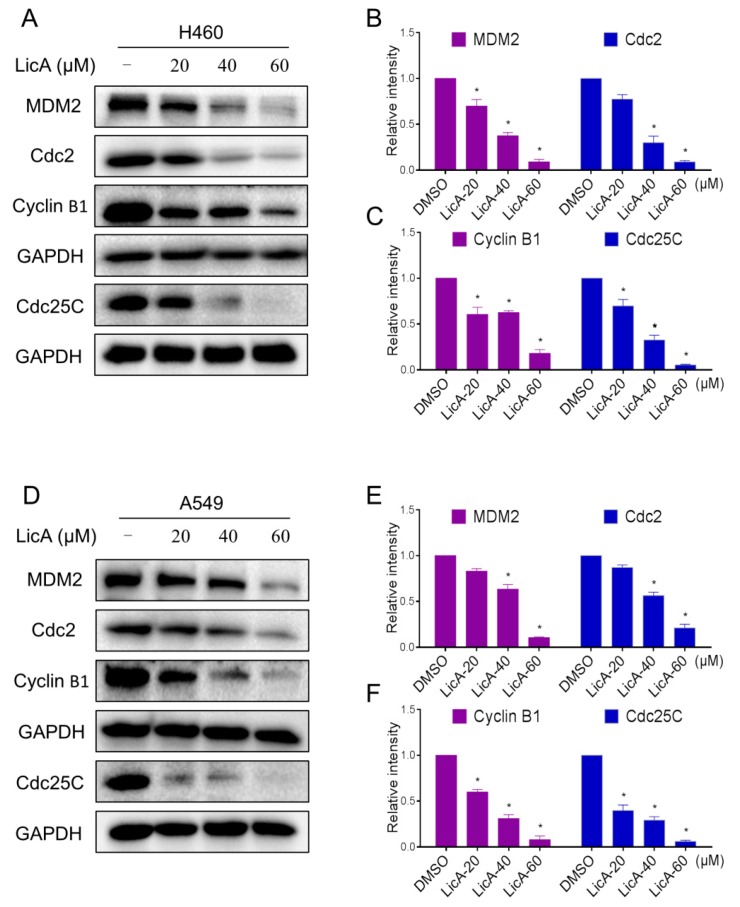
LicA inhibits G2/M cell cycle relative proteins expression. (**A**–**F**) H460 and A549 cells were treated with LicA (20, 40 or 60 μM) for 20 h, the protein levels of MDM2, Cdc2, Cyclin B1 and Cdc25C were determined by Western blot. Western blot results were calculated and represented as the percent of control (* *p* < 0.05). All images shown here are representative of three independent experiments with similar results.

The western blot analysis indicated that treatment with LicA dose-dependently inhibited the expression of MDM2, Cyclin B1, Cdc2 and Cdc25C in H460 and A549 cells (Figure 3A–D). These results indicate that the inhibition of cell proliferation by LicA is partly associated with the induction of G2/M phase arrest in H460 and A549 cells.

### 2.3. LicA Induced Apoptosis in Human Lung Cancer Cells

**Figure 4:**
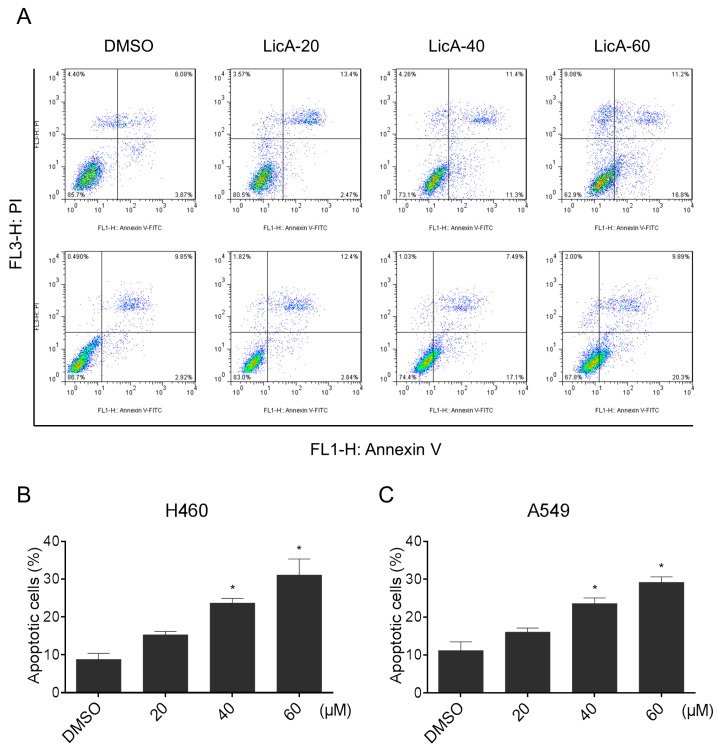
LicA induces apoptosis in human lung cancer cells. (**A**–**C**) H460 and A549 cells were treated with LicA (20, 40 or 60 μM) for 24 h, and the cell apoptosis was analyzed by flow cytometry. Histogram illustrating of the rate of apoptosis cells from FACS analysis of three separate treatments (* *p* < 0.05). All images shown here are representative of three independent experiments with similar results.

We then evaluated the role of apoptosis in LicA-induced cell death using an Annexin V/propidium iodide (PI) staining assay. Results showed that there was a concentration-dependent accumulation of apoptotic cells in LicA-exposured group (Figure 4A–C).

**Figure 5:**
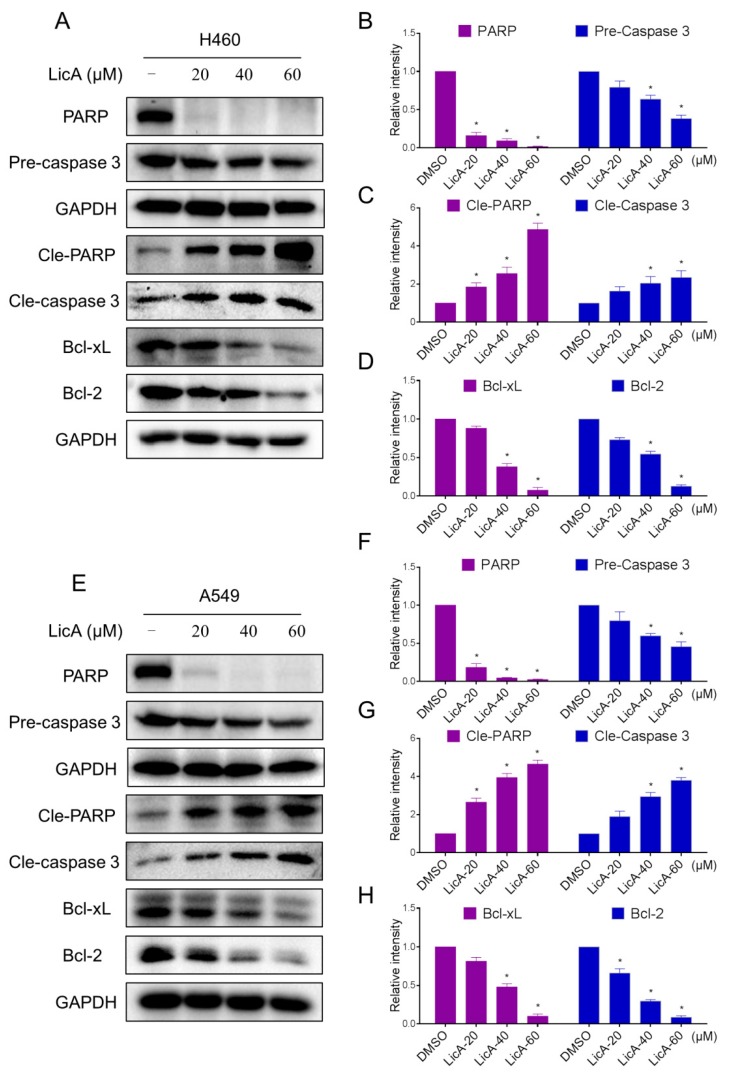
LicA induces apoptosis relative proteins expression. (**A**–**H**) H460 and A549 cells were treated with LicA (20, 40 or 60 μM) for 20 h, the protein levels of PARP, Pre-caspase 3 Cle-PARP, Cle-caspase 3, Bcl-2 and Bcl-xL were determined by Western blot. Western blot results were calculated and represented as the percentage of control (* *p* < 0.05). All images shown here are representative of three independent experiments with similar results.

The levels of apoptosis-associated proteins were also examined by Western blot analysis. As shown in Figure 5A–H, the levels of cleaved PARP and cleaved caspase-3 were increased, and antiapoptotic proteins pre-caspase 3, PARP, Bcl-xL and Bcl-2 were decreased after treatment with LicA for 20 h. These results suggest that LicA-induced apoptosis is associated with PARP/Bcl-2 pathway in H460 and A549 cells.

### 2.4. ER Stress Pathway Is Involved in LicA-Induced Apoptosis

**Figure 6:**
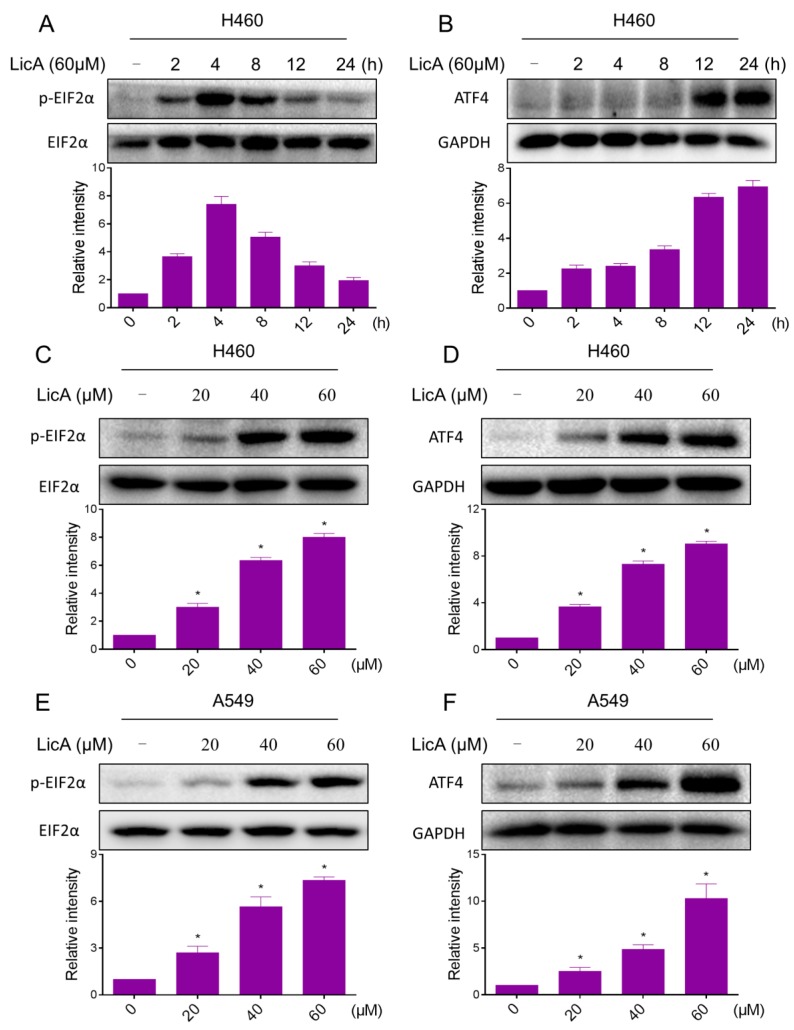
LicA significantly activates the ER stress pathway in human lung cancer cells. (**A**,**B**) H460 and A549 cells were treated with LicA (60 μM) the indicated number of times, and the protein levels of p-EIF2α and ATF4 were determined by Western blot; (**C**,**D**) H460 cells were treated with LicA (20, 40 or 60 μM) the indicated number of times, and the protein levels of p-EIF2α (treatment with LicA for 4 h) and ATF4 (treatment with LicA for 12 h) were determined by Western blot; (**E**,**F**) A549 cells were treated with LicA (20, 40 or 60 μM) the indicated number of times, and the protein levels of p-EIF2α (treatment with LicA for 4 h) and ATF4 (treatment with LicA for 12 h) were determined by Western blot. Western blot results were calculated and represented as the percentage of control (* *p* < 0.05). All images shown here are representative of three independent experiments with similar results.

It is well known that ER stress plays an important role in the initiation of agent-induced apoptosis [25,26]. Therefore, we hypothesize that exacerbation of ER stress contributes to lung cancer cells apoptosis by LicA treatment. We firstly examined the expressions of ER stress-related proteins p-EIF2α and ATF4 in LicA-treated lung cancer cells. The time-course result indicated that LicA (60 μM) could significantly activates ER stress. The protein levels of p-EIF2α and ATF4 reached the peak at 3–6 h after LicA treatment (Figure 6A,B). LicA also increased the expression of these two proteins in a dose-dependent manner (Figure 6C–F). These results suggest that ER stress pathway may potentially be involved in LicA-induced lung cancer cell apoptosis.

## 3. Discussion

Natural products have historically been a critical source of anti-cancer agents, and some of them are currently used in clinical practice [27,28]. Natural products are widely studied as potential therapeutic agents because they are highly effective and less toxicity. LicA is a novel estrogenic flavonoid and a natural phenol product. In this study, we found that LicA significantly induced cell cycle arrest and cell apoptosis in two lung cancer cell lines, accompanied with the corresponding cell cycle and apoptosis-related proteins expression changes.

A growing body of evidence has reported that mitochondria play an important role in apoptosis induced by many agents [14,25]. The Bcl2 family of intracellular proteins is the central regulator of caspase activation. The Bcl-2 family of proteins comprises the anti-apoptotic proteins such as Bcl-xL and Bcl-2, and pro-apoptotic proteins such as Bad and Bax. Bcl-2 family proteins could localize to mitochondria and regulate the mitochondrial membrane potential in response to apoptotic stimulation [29]. In this study, treatment with LicA significantly decreased Bcl-2 and Bcl-xL expression. The imbalance of Bcl-2 family expression finally resulted in the apoptosis of H460 and A549 cells.

ER stress-induced cancer cell apoptosis becomes a critical signaling target for development of cancer therapy drugs. Apoptosis is primarily induced via the “intrinsic pathway”, and the inductions of cancer cell apoptosis by some anti-cancer agents such as piperlongumine and auranofin have been reported to be mediated by ER stress. [13,25]. Targeting ER stress can be a useful anti-cancer strategy. Recently, it was reported that some compounds exerts its proapoptotic effects by inducing ER stress in H460 and A549 cells [30,31,32]. In the present study, we observed an increasing of ER stress-related proteins such as ATF4 and p-EIF2α in a time- and dose-dependent manner, indicating that ER stress was activated after LicA treatment.

In conclusion, we investigated the anti-proliferative effects and mechanisms of LicA in lung cancer cell lines. We found that LicA exhibited antitumor effects by inducing cell cycle arrest and apoptosis. LicA treatment resulted in ER stress activation, which in turn induced apoptotic cell death. Taken together, these results indicate that the LicA possesses great potential as a promising candidate for the treatment of lung cancer. In addition, we also demonstrated that ER stress activation could be an important strategy for the development of new anti-cancer drugs.

## 4. Materials and Methods

### 4.1. Cell Culture and Reagents

Human NSCLC cell line A549 and H460 were purchased from the Institute of Biochemistry and Cell Biology, Chinese Academy of Sciences (Shanghai, China). The cells were routinely cultured in RPMI 1640 medium (Gibco, Eggenstein, Germany) containing 10% heat-inactivated fetalbovine serum (Gibco, Eggenstein, Germany), 100 units/mL penicillin, and 100 ug/mL streptomycin in a humidified cell incubator with an atmosphere of 5% CO_2_ at 37 °C. Antibodies including anti-MDM2 (sc-965, 1:200), anti-Cdc2 (sc-54, 1:200), anti-Cdc25C (sc-13138, 1:200) anti-Cyclin B1 (sc-245, 1:200), anti-Bcl-2 (sc-7382, 1:200), anti-Bcl-xL (sc-7382, 1:200), anti-cleaved PARP-1 (sc-56196, 1:200), anti-GAPDH (sc-293335, 1:1000), goat anti-mouse IgG-HRP and donkey anti-rabbit IgG-HRP were purchased from Santa Cruz Biotechnology (Santa Cruz, CA). Antibodies including anti-ATF4 (#11815, 1:1000), anti-p-EIF2α (#3398, 1:1000) and anti-EIF2α (#9722, 1:1000) were purchased from Cell Signaling Technology (Danvers, MA, USA).

MTT (3-[4,5-dimethylthiazol-2-yl]-2,5-diphenyltetrazolium bromide), Licochalcone A and DMSO (dimethyl sulfoxide) were purchased from Sigma (St. Louis, MO, USA); FITC Annexin V apoptosis Detection Kit I and PI (propidium iodide) were purchased from BD Pharmingen (Franklin Lakes, NJ, USA).

### 4.2. Cell Viability Assay

Cells were seeded into 96-well plates at a density of 5 × 10^3^ per well for 24 h and then treated with LicA, and DMSO was used as the vehicle control. LicA was dissolved in DMSO and diluted with 1640 medium to final concentrations of 2.5, 5, 10, 20, 30, 40, 50, 60 and 80 μM. Cells were incubated with LicA for 24 or 48 h before the MTT assay.

### 4.3. Cell Cycle Analysis

A549 and H460 cells were seeded in 6-well plates for 12 h, and then treated with LicA (20, 40 or 60 μM) or vehicle (DMSO) for 16 h. Cells were collected, then fixed with 75% ice-cold ethanol and stored at −20 °C for 1 h. After centrifugation, the cells were washed with ice-cold PBS twice, then stained with PI at 4 °C for 20 min in the dark. Cell cycle analysis was performed in an FACS Calibur flow cytometer. The cell fractions in the G2/M phase were used for statistical analysis using the FlowJo 7.6 software (TreeStar, San Carlos, CA, USA).

### 4.4. Cell Apoptosis Analysis

A549 and H460 cells were seeded in 6-well plates for 12 h, and then treated with LicA (20, 40 or 60 μM) or vehicle (DMSO) for 24 h. Cells were then harvested, washed twice with ice-cold PBS. The washed cell samples were incubated with Annexin-V for 10 min in the dark and then incubated with PI for 5 min, then evaluated for apoptosis using a FACS Calibur flow cytometer (BD Biosciences, CA, USA).

### 4.5. Western Blot Assay

A549 and H460 cells were seeded in 6-well plates for 12 h, and then treated with LicA (20, 40 or 60 μM) or vehicle (DMSO) for the indicated times. Cells were lysed in lysis buffer, and the lysates were clarified by centrifugation (12,000× *g*) at 4 °C for 10 min. The protein concentration was determined and balanced with purified water. The proteins were separated by 12% sodium dodecyl sulfate-polyacrylamide gel electrophoresis and then transferred to polyvinyldene fluoride membranes. The membranes were blocked using 5% nonfat milk at room temperature for 2 h and then incubated with primary antibodies at 4 °C overnight. Then, the membranes were washed thrice with TBST and incubated with secondary horseradish peroxidase-conjugated antibody for 1 h at room temperature. Finally, the immunoreactive bands were visualized using an ECL kit (Bio-Rad Laboratories, Hercules, CA, USA).

### 4.6. Statistical Analysis

All experiments were assayed in three independent experiments (*n* = 3). The data are expressed as means ± SEM. All statistical analysis were conducted using GraphPad Prism version 5.0 (GraphPad, SanDiego, CA, USA). Two-way ANOVA and Student’s *t*-test were employed to analyze the differences between sets of data. A *p* value < 0.05 was considered statistically significant.

## 5. Conclusions

Taken together, our results indicate that the LicA possesses great potential as a promising candidate for the treatment of lung cancer.
